# *MC4R* mutant mice develop ovarian teratomas

**DOI:** 10.1038/s41598-021-83001-w

**Published:** 2021-02-10

**Authors:** Abdullah An Naser, Takehiro Miyazaki, Jun Wang, Shuji Takabayashi, Theeranukul Pachoensuk, Toshinobu Tokumoto

**Affiliations:** 1 Integrated Bioscience Section, Graduate School of Science and Technology, National University Corporation Shizuoka University, Ohya 836, Suruga-ku, Shizuoka, 422-8529 Japan; 2 Department of Bioscience, Faculty of Science, Shizuoka University, Shizuoka, 422 Japan; 3 Laboratory Animal Facilities & Services, Preeminent Medical Photonics Education & Research Center, Hamamatsu University School of Medicine, 1-20-1, Handayama, Higashi-ku, Hamamatsu, Shizuoka 431-3192 Japan; 4 Present Address: Department of Molecular Genetics, Graduate School of Medicine, Kyoto University, Yoshida Konoe, Sakyo, Kyoto, 606-8501 Japan

**Keywords:** Cancer, Cell biology, Developmental biology, Genetics, Molecular biology, Oncology

## Abstract

Teratomas in mice, composed of different tissue types, are derived from primordial germ cells (PGCs) in the foetal gonads. The strongest candidate gene in the testicular teratoma locus (*Ter*) responsible for testicular teratoma formation was identified as mutation in Dnd1, Dnd1R178*. However, the phenotype of mice with a mutated *Dnd1* gene was germ cell loss. This suggests that other genes are involved in teratoma formation. Testicular teratomas can also be induced experimentally (experimentally testicular teratomas: ETTs) in 129/Sv mice by transplanting E12.5 foetal testes into adult testes. Previously, we mapped the *ett1* locus, which is the locus responsible for ETT formation on chromosome 18. By exome sequence analysis of the 129 and LTXBJ (LT) strains, we identified a missense mutation in the *melanocortin 4 receptor (MC4R)* gene among 8 genes in the *ett1* region. The missense mutation causes a substitution of glycine 25 by serine. Thus, this gene is a candidate for ETT formation. We established the LT-*ett1* congenic strain, which introduced the locus responsible for ETT formation genetically into the genomes of a testicular teratoma non-susceptible strain. In this study, we crossed LT-*ett1* and a previously established LT-*Ter* strain to establish the double congenic strain LT-*Ter*-*ett1*. Also, we established a strain with a point mutation in the *MC4R* gene of the LT strain by genome editing, LT-*MC4R*^*G25S*^. Furthermore, double genetically modified strain LT-*Ter-MC4R*^G25S^ was established to address the relation between *Ter* and *MC4R*. Surprisingly, highly developed ovarian teratomas (OTs), instead of testicular teratomas, appeared not only in the LT-*Ter-MC4R*^*G25S*^ and LT-*MC4R*^*G25S*^ strains but also in the LT-*ett1* and LT-*Ter*-*ett1* strains. The incidence of OT formation was high in double genetically modified strains. The results demonstrated that *MC4R* is one of the genes responsible for OT formation. It was suggested that the effect of the missense mutation in *MC4R* on teratoma formation was promoted by abnormal germ cell formation by the mutation in *DND1.*

## Introduction

Mouse testicular teratomas are composed of various tissues and are derived from primordial germ cells (PGCs) in foetal testes^1^. Strain 129X1/Sv, which is genetically susceptible to testicular teratoma, was established^2,3^. Teratomas are also experimentally inducible by transplanting foetal testes into adult testes in specific strains^4^. To distinguish naturally induced teratomas from experimentally induced teratomas, we referred to them as spontaneous testicular teratomas (STTs) and experimental testicular teratomas (ETTs), respectively. The STT generating ratio is 1–7% in the 129 sublines but almost 100% in 129/Sv (hereafter referred to as 129)-*Ter* mice^5,6^. *Ter* locus identified by D18Mit62 (36.8 Mb) was mapped on chromosome 18^7^. The nonsense mutation which stops at arginine 178 in dead-end 1 (*Dnd1*) gene**,** Dnd1R178*, was identified^8^. *Ter* congenic mice (B6-*Ter* (129/129), LTXBJ (hereafter referred to as LT)-*Ter* (129/129) and C3H-*Ter* (129/129)) do not generate STTs but show the phenotype of PGC loss^6^. However, experimental testicular teratoma generation can be induced in 129-*Ter* (*LT*/*LT*) sublines by transplanting E12.5 foetal testes into adult testes^4^. ETTs do not require the *Ter* mutation. The mice (129-*Ter*^*LT/LT*^) that do not generate STTs can produce ETTs by transplantation^9^. These results indicate that other genes are responsible for testicular teratoma formation^10,11^.

Ovarian teratomas (OTs) develop from female germ cells^12,13^. Contrary to STTs, the molecular mechanisms of OTs are largely unknown. An OT response region was reported on chromosome 6, named *Ots1*, but responsible genes have not yet been identified^14^. However, recent molecular genetic approaches have demonstrated that OTs formation can be induced by various manipulations of genes regulating the activities of follicular cells and oocytes^15^. Follicular cells and oocytes are under the control of many factors, and changes in the quality and quantity of these factors affect the regulation of meiotic arrest and the apoptosis of oocytes or the activity of follicular cells. From the series of results, it is expected that mutations in many of these regulatory genes could cause OTs.

Previously, we conducted linkage analysis using F2 inter-crossed foetuses derived from the F1 of mice susceptible to ETTs, which include 129-*Ter*^*LT/LT*^ mice and those not susceptible to ETTs, LT mouse hybrids. Fine mapping on chromosomes 18 and 19 of the locus associated with generating ETTs demonstrated the locus responsible for ETT formation as “*experimental testicular teratoma 1* (*ett1*)” on chromosome 18^16^.

Also we established the LT-*ett1* congenic strain, the *ett1* region of the LT is replaced with the corresponding region of the 129 strain, whereas all other chromosomes are derived from the LT strain. It is confirmed by transplantation experiments using congenic males homozygous for the *ett1* loci that *ett1* locus contains the gene responsible for ETTs. Eight genes are found in the *ett1* locus which span 1.1 Mb (66.3–67.4 Mb) on chromosome 18. By exome sequencing analysis, we identified a SNP that introduces an amino acid substitution in *melanocortin 4 receptor (MC4R)* among these 8 genes^17^.

In this study, we established a gene-edited strain, LT-*MC4R*^*G25S*^ by using a gene transfer method by electroporation (the TAKE method)^18,19^. To introduce single-nucleotide exchange (G25S) into the *ett1* candidate *MC4R* of the LT strain, we designed a set of guide RNAs for CRISPR/Cas9 and a single-stranded oligodeoxynucleotides (ssODN).

As a result, we succeeded in establishing the desired G25S knock-in mouse, LT-*MC4R*^*G25S*^, in addition to various other mutations in *MC4R*. To explore the roles of *MC4R*^*G25S*^ in ETT and STT formation, we established LT-*Ter*-*MC4R*^*G25S*^ by crossing the LT-*MC4R*^*G25S*^ and LT-*Ter* strains. We also established LT-*Ter*-*ett1* by crossing the LT-*ett1* and LT-*Ter* strains. Surprisingly, highly developed ovarian teratomas (OTs) were formed in the females of these strains. The results demonstrated that *MC4R* is one of the genes responsible for ovarian teratoma formation.

## Results

**Figure 1:**
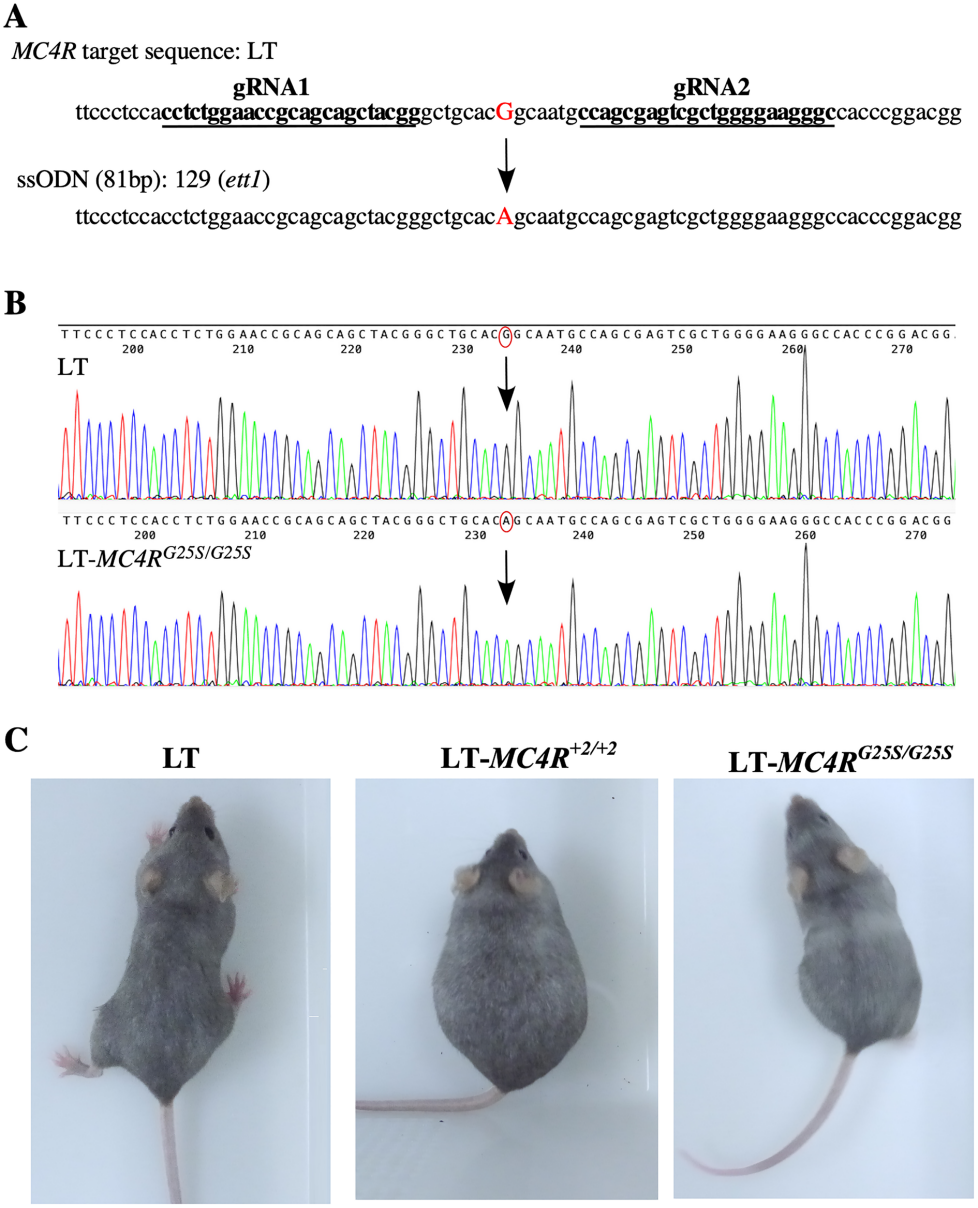
Gene editing of *MC4R* in the LT strain. (**A**) Design of gRNAs to induce single-nucleotide exchange in *MC4R*. Sequences of gRNAs within the target sequence for genome editing are indicated by underlines. The sequence of a single-stranded oligodeoxynucleotide (ssODN) corresponding to the 129 strain is indicated below. The target nucleotides exchanged are indicated in red. (**B**) The results of DNA sequencing analysis of LT and LT-*MC4R*^*G25S/G25S*^ strains are indicated. The target nucleotides exchanged are indicated by red circles. (**C**) Photographs of LT, LT-*MC4R*^+*2/*+*2*^ and LT-*MC4R*^*G25S/G25S*^ female mice.

Genome editing to introduce the identified single-nucleotide substitution in *MC4R* of the LT strain to the 129 strain was performed by using the CRISPR/Cas9 system. Two gRNAs (gRNA1, gRNA2) flanking the target site were selected by CRISPRdirect (https://crispr.dbcls.jp). A ssODN was designed to have a 40 bp homologous sequence at each end of the target site (Fig. 1A).

TAKE (Technique for Animal Knockout system by Electroporation) was used as the introduction method^18,19^. This method does not require the injection of a reagent into a fertilized egg and enables easy genome editing.

In total, 110 2-cell stage embryos that were transfected with the CRISPR/Cas9 system were implanted, resulting in the birth of 13 offspring (11.82%). Among the 13 juveniles, 10 were F0 generation mice in which some mutation was introduced (76.92%). DNA sequence analysis of the F1 generation from the crossing of F0 and wild-type LT strain showed five types of mutations in addition to the desired knock-in mutation to substitute G in LT to A in 129 to induce *MC4R*^*G25S*^ (Fig. 1B). Mutations with a 2 bp insertion (+ 2), a 1 bp deletion (Δ1), a 26 bp deletion (Δ26), and a 27 bp deletion (Δ27) were obtained (Supplementary Fig. S1). + 2, Δ1, and Δ26 are mutations that introduce a stop codon due to frameshifting, and Δ27 causes the deletion of 9 amino acids from the MC4R protein. Homozygous strains for each genotype of + 2, Δ1, Δ26, Δ27 and *MC4R*^*G25S*^ were established. Gene knockout genotype mouse strains (+ 2, Δ1, Δ26) showed an obese phenotype that indicates *MC4R* deficiency, as reported (Fig. 1C). In contrast to the knockout mouse (+ 2, Δ1 and Δ26), knock-in mouse LT-*MC4R*^*G25S/G25S*^ did not show any obvious phenotype (Fig. 1C). Additionally, LT-*MC4R*^*Δ27/Δ27*^ did not show an obese phenotype. We maintained lines LT-*MC4R*^+*2/*+*2*^, LT-*MC4R*^*Δ26/Δ26*^, LT-*MC4R*^*Δ27/Δ27*^ and LT-*MC4R*^*G25S/G25S*^ for further analysis.

**Figure 2:**
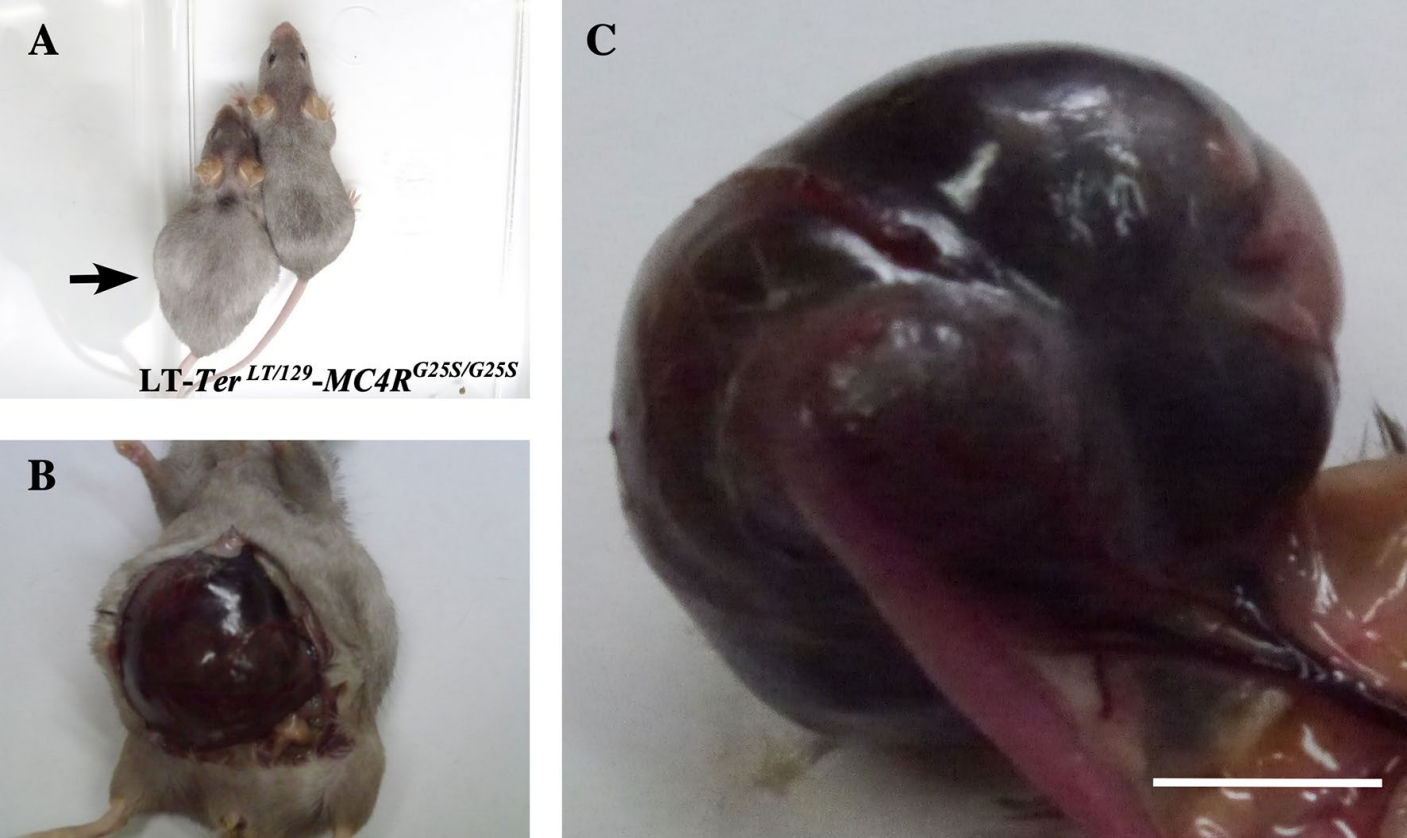
Teratoma formation in the double genetically modified strain LT-*Ter*^*LT/129*^-*MC4R*^*G25S/G25S*^. (**A**) A photograph of females of the LT-*Ter*^*LT/129*^-*MC4R*^*G25S/G25S*^ strain. The mouse on the left side had a swollen abdomen. (**B**) A developed ovarian teratoma was found in the swollen abdomen upon dissection. (**C**) An enlarged image of an ovarian teratoma. Scale bar = 1 cm.

We crossed LT-*MC4R*^*G25S/G25S*^ and LT-*Ter* mice to address whether the substitution of amino acids in *MC4R* resulted in the formation of STTs. However, we found that some LT-*Ter*-*MC4R*^*G25S/G25S*^ females had swollen abdomens at approximately 3 months old or older. We found highly developed OTs after dissection in LT-*Ter*^*LT/129*^-*MC4R*^*G25S/G25S*^ mice (Fig. 2A–C). These results suggested that Dnd1R178* mutation in *Ter* region and *MC4RG25S* mutation cooperatively induce OTs.

**Figure 3:**
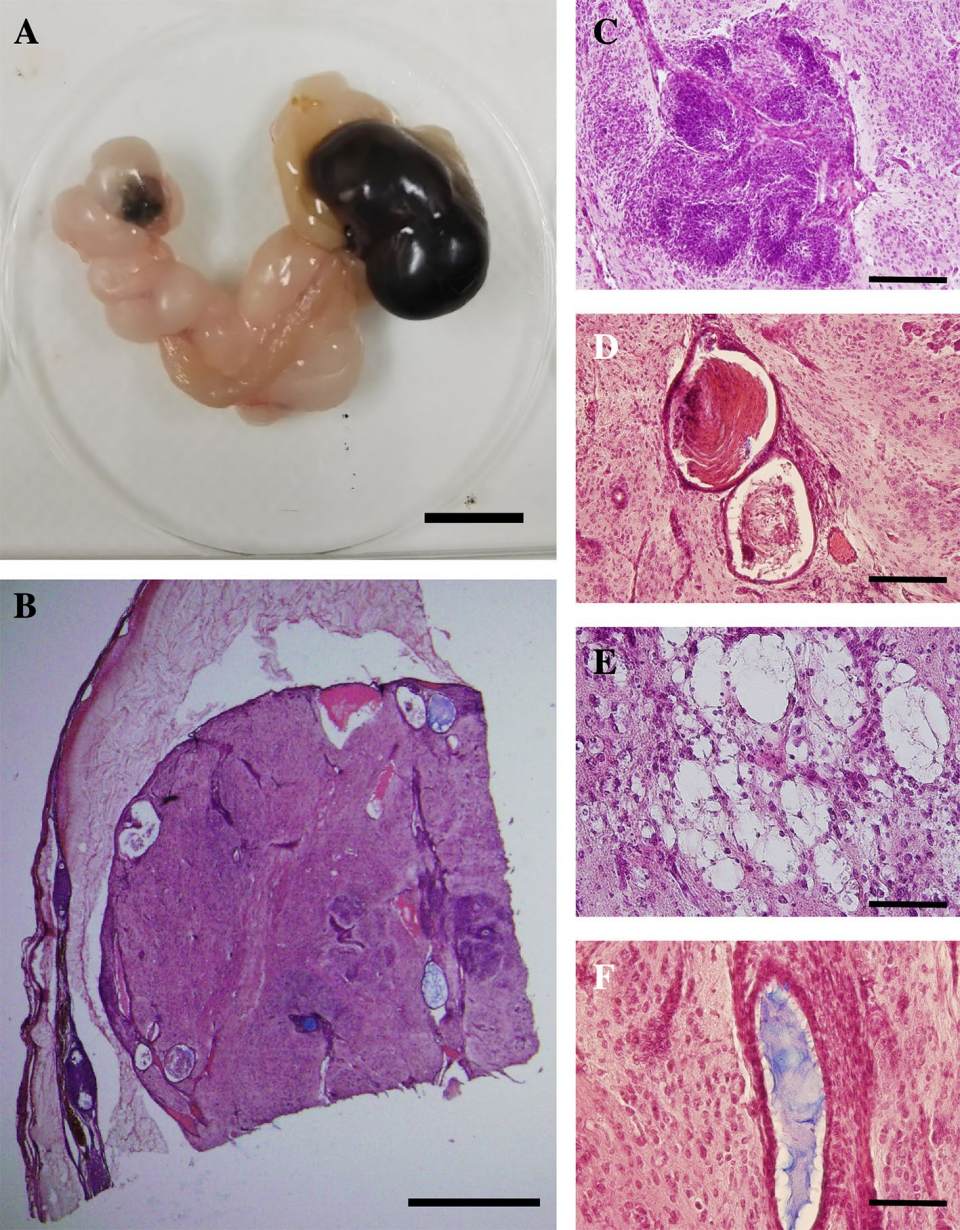
Teratoma formation in the knock-in strain LT-*MC4R*^*G25S/G25S*^. (**A**) An ovarian teratoma found in a female of the LT-*MC4R*^*G25S/G25S*^ strain. Scale bar = 1 cm. (**B**) A representative histological section of a developed ovarian teratoma. Scale bar = 1 cm. (**C**–**E**) Photographs of tissue-like structures found in a teratoma: (**C**) neuronal tissue; (**D**) keratin pearl; (**E**) adipose tissue; (**F**) mucinous glandular structure with ciliated epithelium. Scale bars = 200 μm.

Then, we checked the ovaries of LT-*MC4R*^*G25S/G25S*^ mice. OTs were also found in LT-*MC4R*^*G25S/G25S*^ mice (Fig. 3A). In OTs, various types of tissues of three germ layers, such as neuronal tissue, keratin pearl (ectodermal), adipose tissue (mesodermal), and mucinous glandular structure with ciliated epithelium (endodermal), were observed (Fig. 3B–F).

We also crossed LT-*Ter* and LT-*ett1* mice to establish an LT-*Ter*-*ett1* double congenic strain to address genetic changes in the *ett1* region in combination with *Ter* mutation, resulting in the formation of STTs**.**

**Figure 4:**
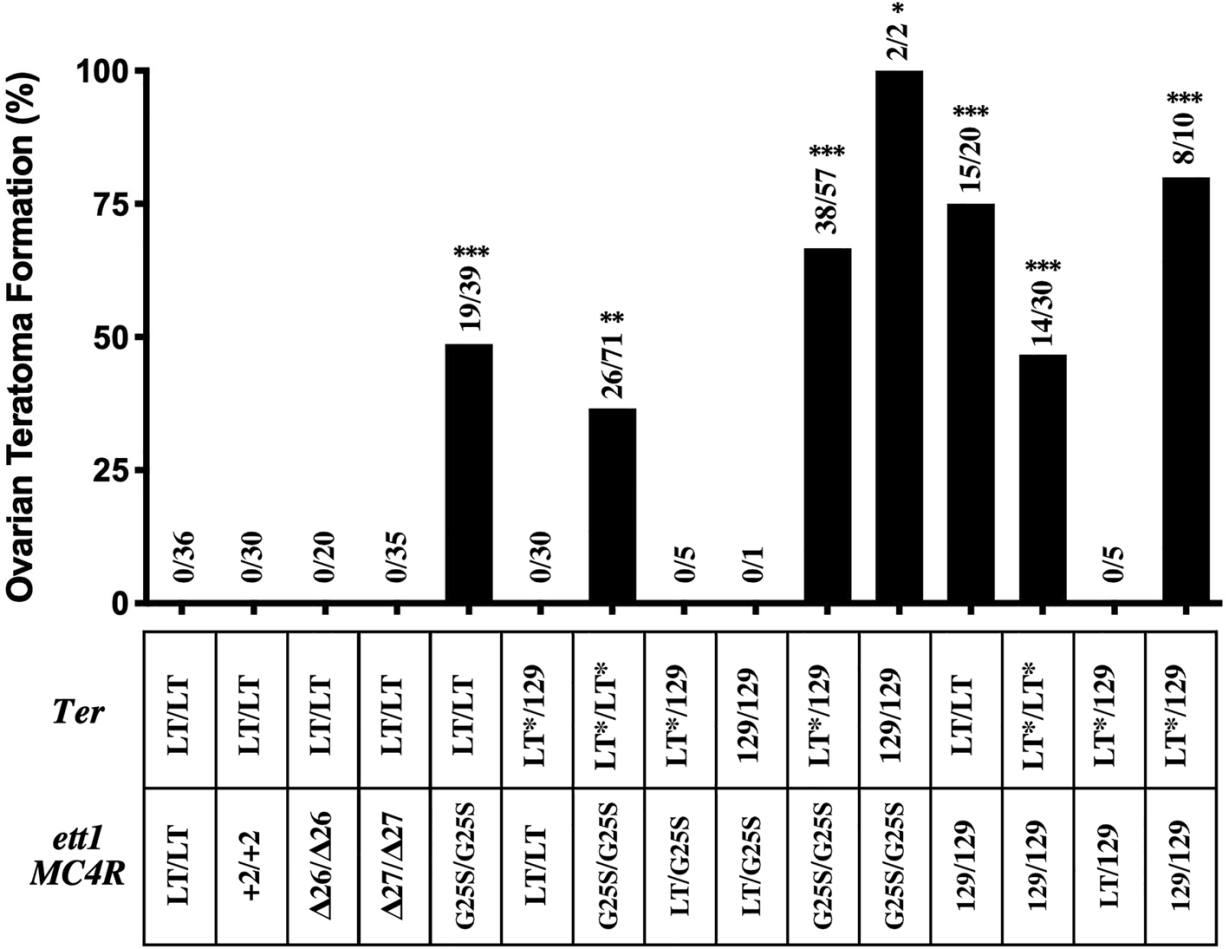
Incidence of ovarian teratoma formation in various strains. The genetic background of all the strains is LT. Numbers on columns represent numbers of teratoma-developing mice among the total numbers of mice checked. The table under the bars represents the genotypes of each strain. Upper row indicates the exchange of the *Ter* region between strain 129. Lower row indicates changes in the *ett1* region (insertion, deletion or knock-in in the *MC4R* gene or the exchange of the *ett1* region). LT* indicate an insertion of chromosome derived from 129 strain identified by D18Mit84 (Supplementary Table S1). *, **, *** indicate significant differences between the value of LT at the P < 0.05, < 0.001, < 0.0001, respectively.

After establishing both the LT-*Ter*-*MC4R*^*G25S*^ and the LT-*Ter*-*ett1* strains, we started to check the ovaries and testes of all the strains for the formation of OTs and STTs. Morphologies of teratomas were photographed and are shown in Supplementary Figs. S2 and S3. The incidents of ovarian teratoma in each genotype are summarized in Fig. 4.

LT-*MC4R*^G25S/G25S^ and LT-*ett1*^*129/129*^ homozygous mutant mice developed OTs, but heterozygous *MC4R*^*LT/G25S*^ and *ett1*^*LT/129*^ mice did not. Although only two survivors of LT-*Ter*^*129/129*^-*MC4R*^*G25S/G25S*^ were obtained, OT was found in both of them (100%). No double-congenic mouse, LT-*Ter*^*129/129*^-*ett1*^*129/129*^, was obtained. The incidence of OTs was significantly higher in LT-*ett1*^*129/129*^ mice (75%) than in LT-*MC4R*^G25S/G25S^ mice (48.7%). The result suggests that the *ett1* region contains genes other than MC4R that contribute to OT formation. The incidence of OTs was significantly increased to 66.7% when the *Ter* region was introduced in heterozygous to *MC4R*^G25S/G25S^ mice. In the case of LT-*ett1*^*129/129*^, the incidence of OTs was not changed by the introduction of *Ter* but was lowered in LT-*Ter*^LT*/ LT*^-ett1^*129/129*^ mice. Also the incidence of OT formation was decreased in LT-*Ter*^LT*/ LT*^-MC4R^*G25S/G25S*^ mice compared with LT-*MC4R*^*G25S/G25S*^ mice. As indicated in Supplementary Table S1, the small region of chromosome 18 in 129 strain identified by the D18Mit84 marker was introduced into LT. Thus, a small fragment of the 129 strain was inserted in LT-*Ter *^LT*/LT*^. There is a possibility that other genes that prevent teratoma formation exist in this region. No OT formation was found in the original strain LT or in mutants of *MC4R* (+ 2, Δ26 and Δ27).

**Figure 5:**
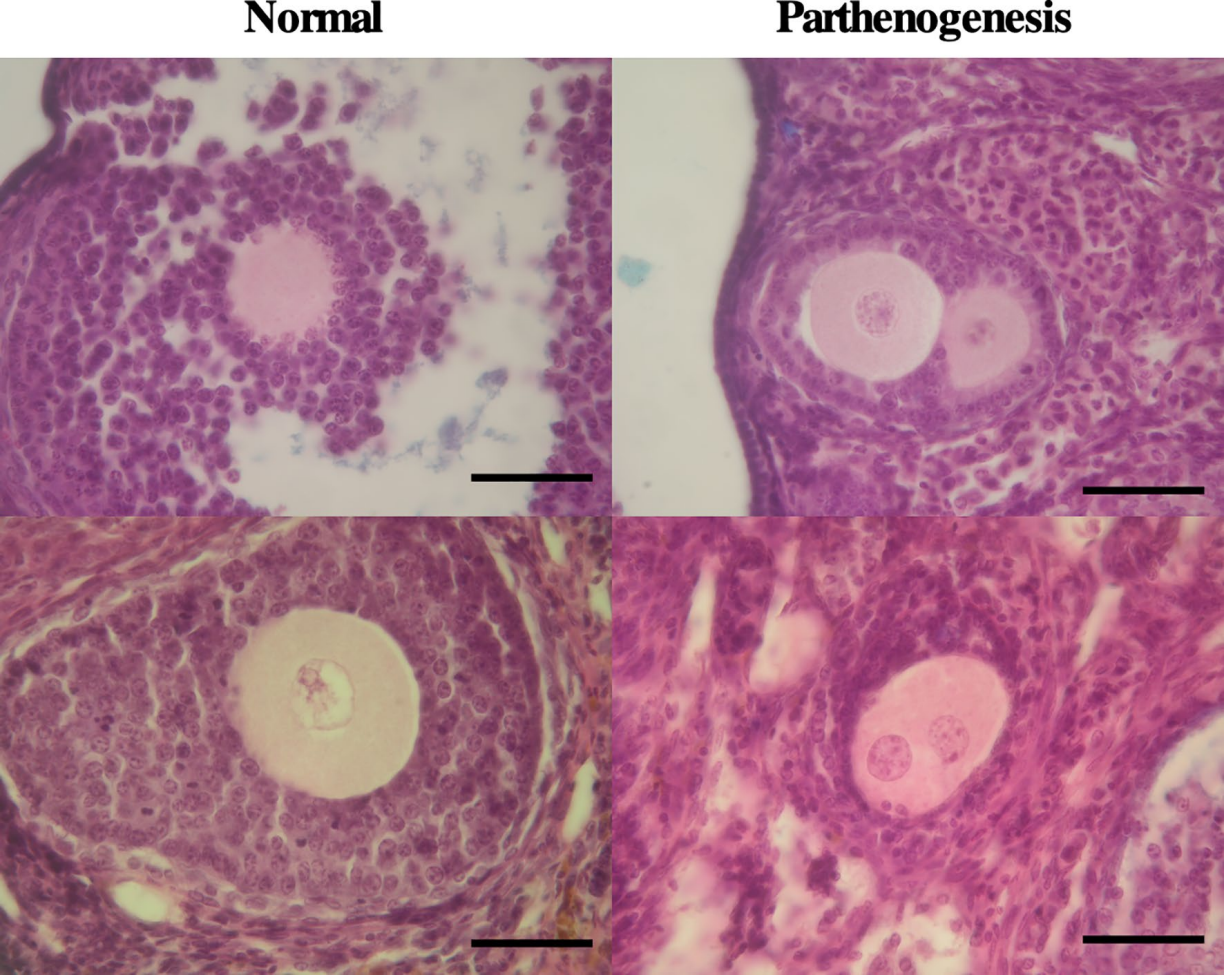
Parthenogenesis of oocytes in the *MC4R* knock-in strain. Representative sections of ovaries from one-month-old females of the LT-*MC4R*^*G25S/G25S*^ strain. Normal oocytes in developed follicles are indicated on the left side. Two cell stage blastomeres in small follicles are shown on the right side. Scale bars = 100 μm.

OTs have been known to develop from the parthenogenesis of oocytes^20,21^. Thus, we checked for this by observing sections prepared from mice that were expected to start to produce teratomas. We prepared sections of ovaries from one-month-old mice. As expected, oocytes divided into 2 cells in the small follicle were found in LT-*MC4R*^*G25S/G25S*^ mice (Fig. 5). One to three 2 cells found in whole ovaries before forming OT (1.6% of primary follicles in three ovaries from three different females, 2 ± 0.6 2 cells/ 127.7 ± 7.5 primary follicles).

**Figure 6:**
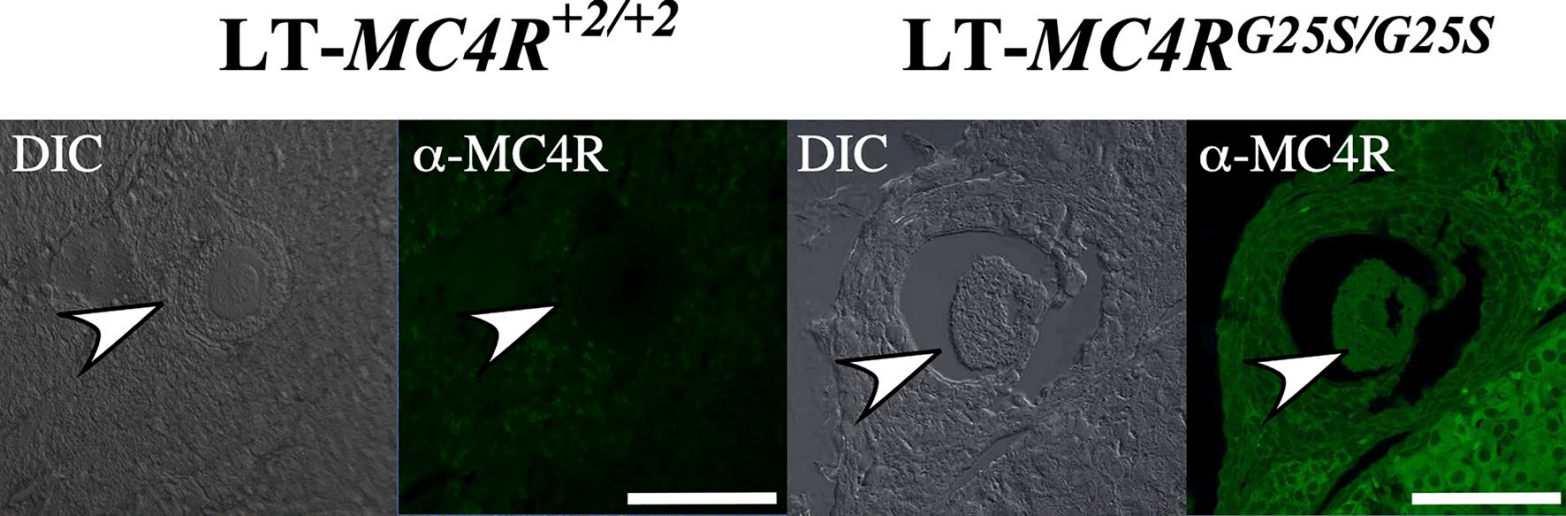
MC4R is expressed in oocytes. Immunohistochemical staining results of sections of ovaries from one-month-old females of the LT-*MC4R*^+*2/*+*2*^ and LT-*MC4R*^*G25S/G25S*^ strain. Sections were stained with anti-rabbit MC4R polyclonal antibodies (α-MC4R). Differential contrast (DIC) images also indicate. Oocyte is indicated by white arrowhead. Scale bars = 100 μm.

It is suggested that these 2 cells are produced by the parthenogenesis of the oocyte. It was demonstrated by immunohistochemical analysis that MC4R proteins were expressed in oocytes of LT-*MC4R*^*G25S/G25S*^, but not in oocytes of LT-*MC4R*^+*2/*+*2*^ mice (Fig. 6).

## Discussion

We introduced the *Ter* gene, which is one of the causative genes for testicular teratoma formation found in 129 strains that develop STTs and ETTs, into LT strains that do not develop STTs and ETTs. A single-nucleotide substitution was introduced into the *MC4R* gene to introduce the same amino acid substitution as in the 129 strain. The introduction of the *Ter* gene resulted in a germ-cell-loss phenotype in both males and females, as in the case of the *Ter*-gene-introduced lines produced from other lines^9^. Then, it was revealed that the *Ter* gene product DND1 is essential for germ cell formation and that other genes are also necessary for the onset of teratomas. We mapped the *ett1* region, which is a candidate region for ETTs, and found a SNP in the *MC4R* gene that causes an amino acid change between the 129 strains and the LT strains among the 8 genes existing in the *ett1* region^17^. Thus, the single amino acid change in the *MC4R* gene is supposed to be a candidate for *ett1*. Then, to identify the *ett1* gene, a knock-in strain, LT-*MC4R*^*G25S/G25S*^, in which this single base substitution was introduced into the LT strain, was created by genome editing. By crossing this knock-in line with the LT-*Ter* line, we tried to investigate the possibility of developing STTs or ETTs due to the combined effect of *Ter* and *MC4R*^*G25S*^. Surprisingly, highly developed OTs developed at an extremely high rate (48.7%). The original LT strain was established as a susceptible strain for OTs, but at the end of 30 years of passage, the current strain has changed so that OTs do not develop at all. At least one and a half years after starting the establishment of the double-gene-modified strain of LT-*Ter*-*MC4R*^*G25S*^ mice, no case of OTs was found in the original strain LT during the observations over four generations. Thus, we introduced two candidate teratoma-causing genes into a line that does not develop OTs and analysed their combined effects. From many years of research so far, the mechanism of ovarian teratoma development is that some of the germ cells begin parthenogenesis for some reason in the process of germ cell apoptosis^13^. Our results suggest that *MC4R* is involved in the regulation of germ cell apoptosis and that changes in the *MC4R*-mediated signal transduction system cause germ cell parthenogenesis.

The *MC4R* gene is involved in energy intake and energy expenditure^22^. MC4R coupled with stimulating G protein (Gs) promotes protein kinase A (PKA) activation and induces cAMP production. The signal transduction pathway through *MC4R* suppresses c-Jun N-terminal kinase (JNK)^23^. From the results of *MC4R* gene targeting, it is well known that *MC4R*-gene-deficient mice become obese, and the *MC4R* gene is deeply involved in energy control^24^. Melanocyte-stimulating hormone (MSH), agouti and agouti-related protein (AGRP) are known as ligands for MC4R^25^. 129-Sv-Ay^(Ay/+)^ male mice are known to have a tenfold lower rate of testicular teratoma formation than wild-type mice^26^. Ay is a dominant mutation in the agouti gene and causes ectopic expression of agouti protein and the inhibition of MC1R and MC4R chronic signal transduction systems^27–29^. As an agonist of MC4R, agouti-related (AGRP) has been found to be a related gene of agouti, and it has been reported that AGRP has a 100-fold higher binding affinity to MC4R than agouti^30^. According to the docking model of MC4R and its ligand, the binding of AGRP and MC4R involves the N-termini of MC4R K33, Y35 and D37, and the binding of MSH and MC4R does not involve the N-terminus of MC4R^31^. From this, it can be speculated that the ligand of MC4R in the development of ovarian teratomas in this double-gene-modified line is AGRP. Additionally, the G25S missense mutation of MC4R inhibits the binding to AGRP, which suppresses the action of the MC4R antiapoptotic signal. As indicated in this study, MC4R is expressed in mouse oocytes. It is hypothesized that the modification of the antiapoptotic pathway of oocytes is relatively enhanced to increase the rate of parthenogenesis and cause ovarian teratoma formation.

Detailed analysis of the function of DND1 identified as the *Ter* gene is underway. Yamaji et al*.* indicated that DND1-dependent mRNA destabilization is required for the survival of mouse PGCs and spermatogonial stem cells by suppressing apoptosis^32^. By contrast, there is a report that showed that DND1 protects and maintains germ cell fate^33^. DND1-deficient germ cells transdifferentiate into somatic cells. The translation of nanos1, a determinant of germ cells, requires DND1^34,35^. Recently, it was demonstrated that Nanos2 and Nanos3 are involved in testicular teratoma formation by establishing a double mutant strain with each gene and *Ter*^36^. Thus, a loss-of-function mutation in *DND1* is essential for teratoma formation.

The OT-related locus was reported previously; however, a mutated gene in the *Ots1* locus has not yet been identified^14^. However, the genes suggested to be responsible for OTs were found with gene knockout approaches. As a result of the generation of many genetically modified mice so far, it has been reported that ovarian teratomas also develop by different mechanisms. The onset of OTs due to parthenogenesis has been reported in mice in which the Mos gene has been knocked out, which has the function of meiotic arrest^21^. Yang et al*.* showed that conditional knockout mice of retinoblastoma protein 1 (*Rb1*) developed ovarian teratomas and that abnormalities of somatic follicular cells also cause teratoma formation^15^. Similarly, the overexpression of *Bcl-2*, constitutive active mutation in the FSH receptor, a missense mutation in *Foxo3a* and the Tgkd transgene insertion mutation in the Inpp4b gene are other examples of teratoma formation caused by somatic abnormalities and the genetic background of mouse strains^37–40^. Although the mechanism of teratoma formation is unknown, global knockdown of the Gata4 gene by siRNA also induced the formation of OTs^41^. In this way, it has become clear that there is an onset mechanism caused by germ-cell-derived teratomas and somatic cells. Many gene mutations can cause teratomas because the formation and divisional control of germ cells are performed under complicated control of the follicular cells covering them and growth factors in the ovary. To elucidate the whole picture of the mechanism of teratoma formation, it is necessary to analyse it at the whole-genome level.

The LT-*Ter*-*MC4R*^*G25S*^ double-gene-modified line we established was used to elucidate the cause of testicular teratomas. Transplantation studies are currently underway to determine whether MC4R amino acid substitution is responsible for ETTs. Additionally, it is currently unknown whether STTs will develop in the double-gene-modified strain. These results will be published in the future.

## Methods

### Mice

All animals were housed under temperature-controlled conditions and had free access to food and water. All animal procedures were performed per the protocols approved by the Institutional Animal Care and Use Committee of Shizuoka University (29A-8, 2018A-19, 2019A-8, 2020A-7). All experiments were performed in accordance with the guideline of the committee. The study was carried out in compliance with the ARRIVE guidelines^42^.

The 129/Sv-*Ter* and LTXBJ mice^12^, generous gifts from Dr Leroy Stevens (The Jackson Laboratory, Bar Harbor, ME, USA), were bred in the animal facilities at Shizuoka University. The LT males exhibited normal spermatogenesis and generated neither STTs nor ETTs^9^. The STT formation rate of the 129 strain was 1.4%, but the formation rate of ETTs formed by the transplantation of foetal testes was 80–90%^9,23^. Males of the LT strain develop spermatogenesis normally and do not form STTs or ETTs^9^. On the other hand, females of the original LT strain reported developing almost 100% of OTs from parthenogenetic eggs after sexual maturity^13^. The LT strain used as the original strain was established as a strain susceptible to ovarian teratomas, but at the end of 30 years of passage, the current strain has changed so that OTs do not develop at all. At least one and a half years after the establishment of the double-gene-modified strain of LT-*Ter*-*MC4R*^*G25S*^, no OT has been found in the original strain LT during the observations over four generations.

The LT-*ett1* congenic strain was established previously and maintained^16^. The LT-*ett1* strain also exhibited normal spermatogenesis and did not generate STTs but formed ETTs when foetal testes were transplanted into adult testes. In the LT-*ett1* strain, the genome region including the *ett1* locus (which exists at 62.0–70.2 M bp and includes peak markers D18Mit81 and D18Mit184) and a region identified by D18Mit84 (33.8 M bp) were substituted with the region from 129-Sv-*Ter* mice^17^.

LT-*Ter*^*129/129*^ exhibited the germ-cell-loss phenotype in both heterozygous and homozygous mutants. In the LT-*Ter*^*129/129*^ strain, the genome region including the *Ter* locus (which exists at 33.8–46.4 Mbp and includes the *Dnd1* gene) was substituted with the 129/Sv-*Ter* region^43^.

The LT-*Ter*-*ett1* strain was established by crossing these two strains and then back-crossing at least 5 more times with LT-*ett1*^*129/129*^*.*

For the sampling of specimens, the mice were sacrificed by cervical dislocation. All efforts were made to minimize animal suffering.

### Creation of genome-edited mice

Using the TAKE method based on CRISPR/Cas9 system, a change in a single nucleotide was introduced into the LT strain by using a ssODN^18,19^. As gRNA, two gRNAs sandwiching the target site were selected by CRISPRdirect (https://crispr.dbcls.jp) (Fig. 1). An 81-bp ssODN with a 40-bp homologous sequence covering the target region was designed. The synthesis of an ssODN was requested from STAR Oligo (RIKAKEN, Nagoya, Japan). PAGE purification was chosen as the grade of synthesis. Genome-edited mice that could be identified by sequence analysis were back-crossed to LT strain mice. The F1 offspring obtained were also genotyped by sequence analysis and then transferred to sibling mating between the same genotypes.

### Genotyping

Genotyping by PCR-Simple sequence length polymorphism (SSLP) and DNA sequencing were done using genomic DNA extracted from the ear of each mouse. *MC4R* target site was amplified by PCR using a primer set (F: 5′-CCGAACCCAGAAGAGACCAA, R: 5 '-GACCCATTCGAAACGCTCAC). DNA sequencing was outsourced to Fasmac Co., Ltd. The DNA sequence analysis was performed using Codon Code Aligner (http://www.codoncode.com/aligner/) and GENETX-MAC (Ver.14.0.3).

### Observation of ovarian teratomas

The morphology of the ovaries of each mouse after three months of age was observed and photographed after dissection. Whether the teratoma was developed on both sides or on which side when developed on only one side was recorded. The weight of the ovaries was measured.

### Histological analysis

Tissues were dissected and fixed in Bouin's solution, embedded in paraffin, and serially sectioned at 6-μm thickness. Deparaffinized sections were stained with haematoxylin, eosin and alcian blue (H-E-A) and screened for the presence of OTs under a light microscope.

### Immunohistochemical observation

Ovaries were fixed with 4% PFA, dehydrated, cleared, and embedded in paraffin to prepare 5 μm-thick sections. After deparaffinization, the tissue was surrounded with a liquid blocker, Pap Pen (DAIDO SANGYO CO., LTD, Tokyo Japan). Sections were blocked with 3% BSA/TBST. After 3 washes with TBST for 10 min, the primary antibody, anti-MC4R (1:80, Cayman Chemical, 10006355), diluted with 3% BSA/TBST, was reacted overnight at 4 °C. Sections were washed 3 times with TBST for 10 min. Then, the secondary antibody, anti-rabbit IgG (H + L), F(ab')2 fragment (Alexa Fluor 488 conjugate) (1:500, Cell Signaling, #4412), diluted with 3% BSA/TBST, was reacted at room temperature for 1 h. After 3 washes with TBST for 10 min, sections were enclosed with a water-soluble encapsulant. A Carl Zeiss confocal laser scanning microscope (LSM700) was used for fluorescence observation.

### Statistical analysis

OT formed mice and none-formed mice of each strain were binarized and the teratoma incidence of each strain was analyzed by one-way analysis of variance (ANOVA) (Tukey’s multiple comparisons test). ANOVA was performed using GraphPad Prism (San Diego, CA).

## Supplementary Information

Supplementary Information.
