# Supplementary material for: MC4R mutant mice develop ovarian teratomas
Source: Sci Rep. 2021 Feb 10;11:3483. doi: 10.1038/s41598-021-83001-w (PMC7876032; doi:10.1038/s41598-021-83001-w)
Supplement: Supplementary file 1 — Supplementary Information. [file 41598_2021_83001_MOESM1_ESM.docx]

Supplementary Information for

***MC4R* mutant mice develop ovarian teratomas**

Abdullah An Naser^1^, Takehiro Miyazaki^1,^ ^+^, Jun Wang^2^, Shuji Takabayashi^3^, Theeranukul Pachoensuk**^1^**, Toshinobu Tokumoto^1,2^

^1^Integrated Bioscience Section, Graduate School of Science and Technology, National University Corporation Shizuoka University, Ohya 836, Suruga-ku, Shizuoka 422-8529, Japan.

^2^Department of Bioscience, Faculty of Science, Shizuoka University, Shizuoka 422, Japan.

^3^Institute for Experimental Animals, Hamamatsu University School of Medicine

1-20-1, Handayama, Higashi-ku, Hamamatsu, Shizuoka 431-3192, Japan

+Present address: Department of Molecular Genetics, Graduate School of Medicine, Kyoto University, Yoshida Konoe, Sakyo, Kyoto 606-8501, Japan

*Running title*: *MC4R* is an ovarian teratoma responsible gene

* Correspondence and requests for materials should be addressed to T.T. ([tokumoto.toshinobu@shizuoka.ac.jp](mailto:tokumoto.toshinobu@shizuoka.ac.jp))

**Figure caption of Supplementary Table and Figures**

**Supplementary Table. S1** *Ter* and *ett1* loci genotyping in the LT-*Ter*-*ett1^129/129^* double congenic strain. The results of the SSLP analysis using microsatellite markers are summarized.

| Marker | cM | Start (Mbp) | *Ter^LT*/LT*^* | *Ter^LT*/129^* | *Ter*^129/129^ |
| --- | --- | --- | --- | --- | --- |
| D18Mit64 | 4.46 | 6.1 | LT | LT | LT |
| D18Mit84 | 18.21 | 33.8 | 129 | 129 | 129 |
| *Dnd1R178** | 19.46 | 36.8 | LT | LT/129 | 129 |
| D18Mit17 | 21.09 | 39.5 | LT | LT/129 | 129 |
| D18Mit163 | 23.29 | 43.4 | LT | LT/129 | 129 |
| D18Mit235 | 23.80 | 44.7 | LT | LT/129 | 129 |
| D18Mit58 | 24.56 | 46.4 | LT | LT/129 | 129 |
| D18Mit24 | 27.12 | 50.0 | LT | LT | LT |
| D18Mit55 | 28.92 | 53.2 | LT | LT | LT |
| D18Mit123 | 30.12 | 56.0 | LT | LT | LT |
| D18Mit152 | 34.78 | 61.9 | 129 | 129 | 129 |
| D18Mit40 | 37.11 | 63.7 | 129 | 129 | 129 |
| D18Mit81 | 39.53 | 66.5 | 129 | 129 | 129 |
| D18Mit184 | 39.70 | 66.8 | 129 | 129 | 129 |
| D18Mit9 | 42.56 | 68.5 | 129 | 129 | 129 |
| D18Mit33 | 43.49 | 69.7 | 129 | 129 | 129 |
| D18Mit103 | 44.19 | 70.2 | 129 | 129 | 129 |
| D18Mit186 | 45.63 | 72.0 | LT | LT | LT |
| D18Mit49 | 51.27 | 76.0 | LT | LT | LT |
| D18Mit47 | 52.38 | 77.8 | LT | LT | LT |
| D18Mit126 | 52.67 | 78.9 | LT | LT | LT |
| D18Mit4 | 57.53 | 84.1 | LT | LT | LT |

**Supplementary Figure. S1** Genotype details of progeny from *MC4R* genome editing. The positions of sites for gRNA are indicated by lines. (A) Nucleotide alignment shows deletions (dashes) and insertions at the target site in the strains of mutants. The numbers of deleted or inserted bases are indicated. (B) Amino acid alignment shows truncated peptides caused by frame shift mutations or deletions. The numbers of length of amino acids of MC4R proteins produced in strains are indicated.

**
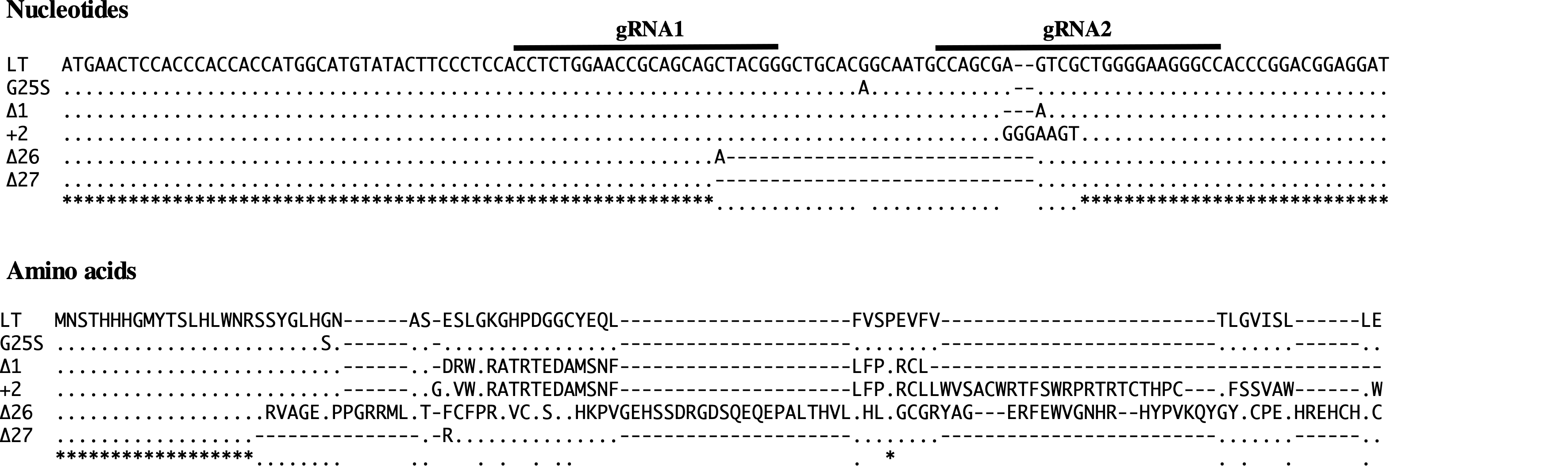
**

**Supplementary Figure. S2**

Morphologies of teratomas found in LT-*MC4R^G25S/G25S^*, LT-*Ter*-*MC4R^G25S^* strains. Females older than three months old or older were dissected and ovaries were excised and photographed. (A) Five specimens from LT-*MC4R^G25S/G25S^*, (B) Five specimens from LT-*Ter^LT*/LT*^-MC4R^G25S/G25S^*, (C) Five specimens from LT-*Ter^LT*129^-MC4R*^G25S/G25S^ and (D) One specimen from LT-*Ter^129/129^-MC4R*^G25S/G25S^. Scale bar = 1cm

**
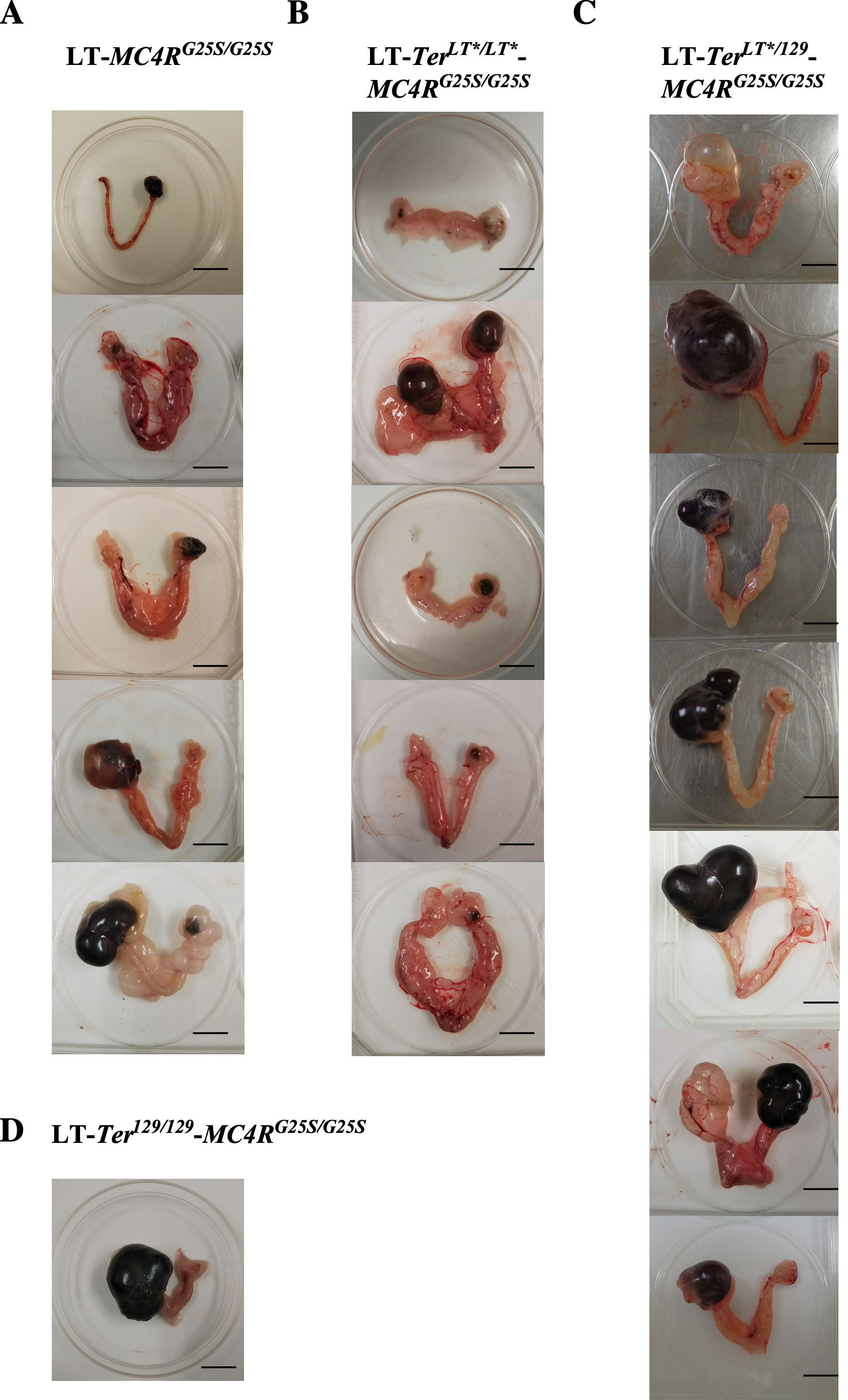
**

**Supplementary Figure. S3**

Morphologies of teratomas found in LT-*ett1^129/129^*, LT-*Ter-ett1^129/129^* strains. Females older than three months old or older were dissected and ovaries were excised and photographed. (A) Three specimens from LT-*ett1^129/129^*, (B) Five specimens from LT-*Ter^LT*/LT*^- ett1^129/129^* and (C) Three specimen from LT-*Ter^LT*/129^-ett1^129/129^*. Scale bar = 1cm.

**
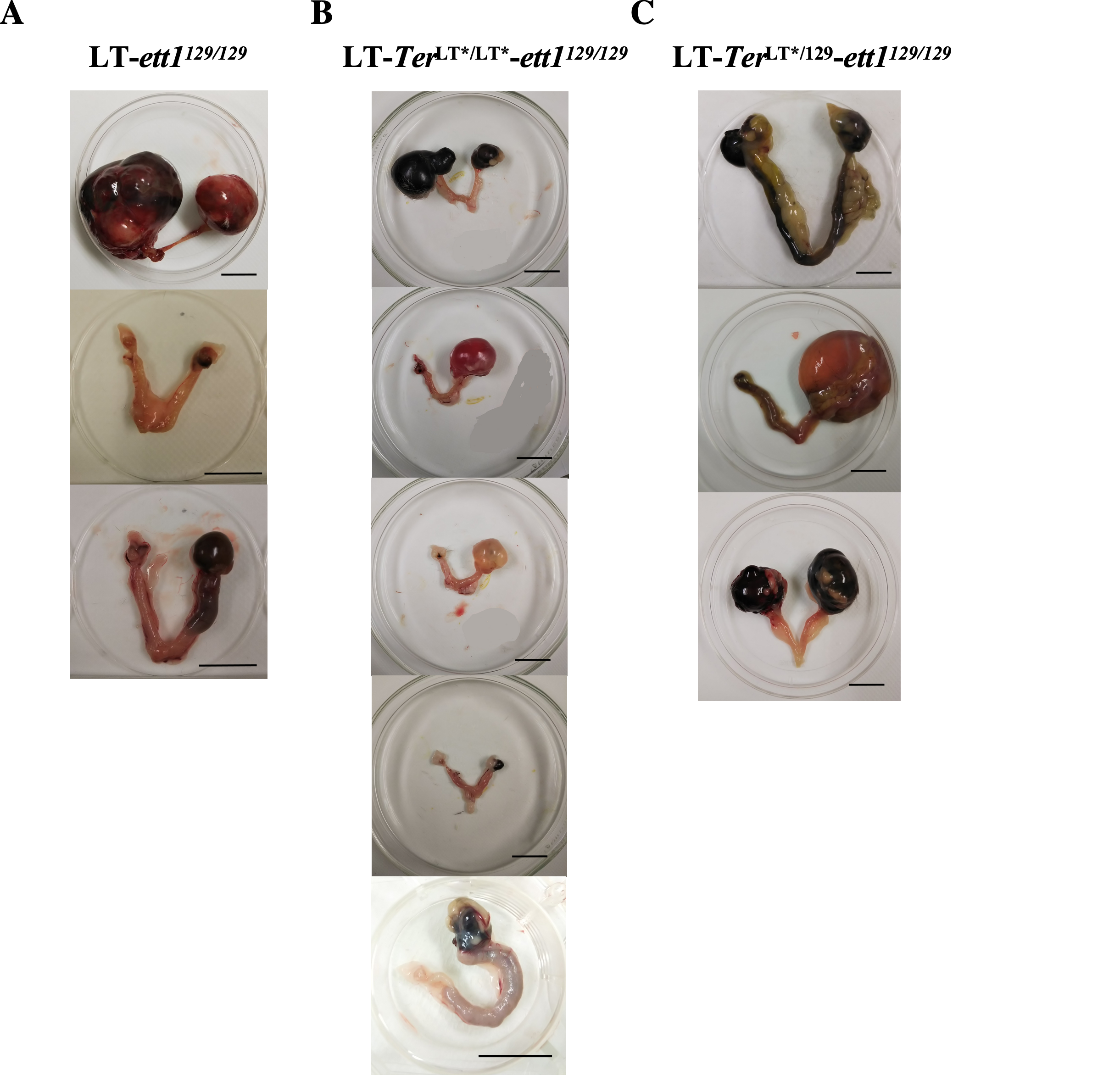
**
